# Reestimation of slab dehydration fronts in Kuril-Kamchatka using updated global subduction zone thermal structures

**DOI:** 10.1016/j.isci.2023.107288

**Published:** 2023-07-11

**Authors:** Weiling Zhu, Yingfeng Ji, Lijun Liu, Rui Qu, Ye Zhu, Chaodi Xie, Lin Ding

**Affiliations:** 1 State Key Laboratory of Tibetan Plateau Earth System, Environment and Resources (TPESER), Institute of Tibetan Plateau Research, Chinese Academy of Sciences, Beijing 100101, China; 2 University of Chinese Academy of Sciences, Beijing 100049, China; 3 University of Illinois at Urbana-Champaign, Urbana, IL, USA; 4 Geophysics Department, School of Earth Sciences, Yunnan University, Kunming 650500, China

**Keywords:** Earth sciences, Geology, Tectonics

## Abstract

Previous subduction thermal models are inconsistent with the values of forearc heat flow (50–140 mW/m^2^) and global P‒T conditions of exhumed rocks, both suggesting a shallow environment 200–300°C warmer than model predictions. Here, we revaluate these problems in Kuril-Kamchatka using 3D thermomechanical modeling that satisfies the observed subduction history and slab geometry, while our refined 3D slab thermal state is warmer than that predicted by previous 2D models and better matches observational constraints. We show that warmer slabs create hierarchical slab dehydration fronts at various forearc depths, causing fast and slow subduction earthquakes. We conclude that fast-to-slow subduction earthquakes all play a key role in balancing plate coupling energy release on megathrusts trenchward of high P-T volcanism.

## Highlights

• Slab thermal state reestimation indicates 200–300°C warmer than previously expected
• Thermally controlled multilayered slab dehydration fronts vary with depths greatly
• High intraslab thermal gradients facilitate the dehydration efficiency below forearc

## Graphical abstract

**Figure:**
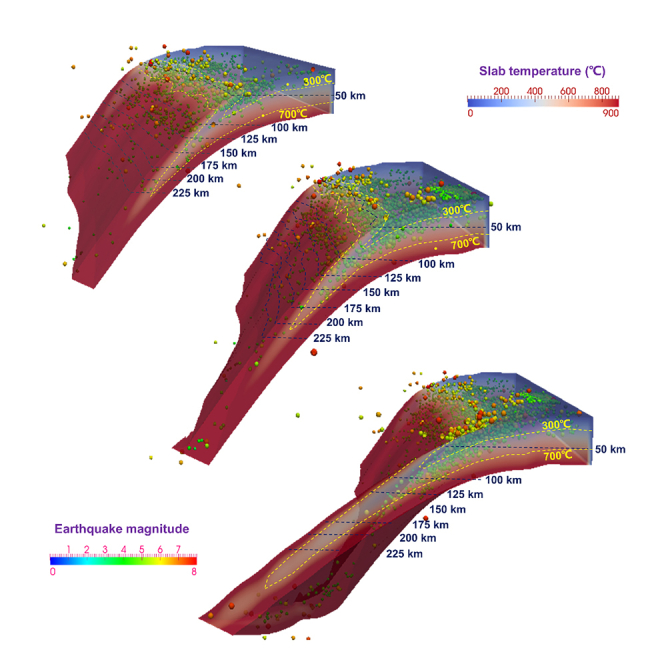

## Introduction

The slab thermal regime is fundamental to subduction zone dynamics, including the budget of slab dehydration and decarbonization, water and mineral circulation, and origins of subduction earthquakes and arc magmatism. Currently, 2D numerical models represent the dominant tool for estimating subduction zone thermal structures^1^ and serve as a basis for many subsequent studies on subduction-related processes.^2^^,^^3^^,^^4^^,^^5^^,^^6^^,^^7^^,^^8^^,^^9^ These models usually assume very low forearc heat flow (<50 mW/m^2^), but observations in the Northeast Japan forearc reveal much higher values (50–140 mW/m^2^) (Figure S1A, e g., Qu et al.^10^). In addition, these 2D model predictions violate the globally observed P-T condition of exhumed rocks, with the latter being 200–300°C warmer than the model predictions at <75 km (or <2.5 GPa) depths (Figure S2, e.g., van Keken et al.^11^). These uncertainties require a revaluation of the true P-T conditions of subduction zones, as well as the associated water flux, carbonate cycling, plate-mantle coupling, earthquake generation, and arc magmatism. In this study, we developed 3D finite difference models to obtain new slab thermal estimates using updated datasets on heat flow and slab geometry.

**Figure 1:**
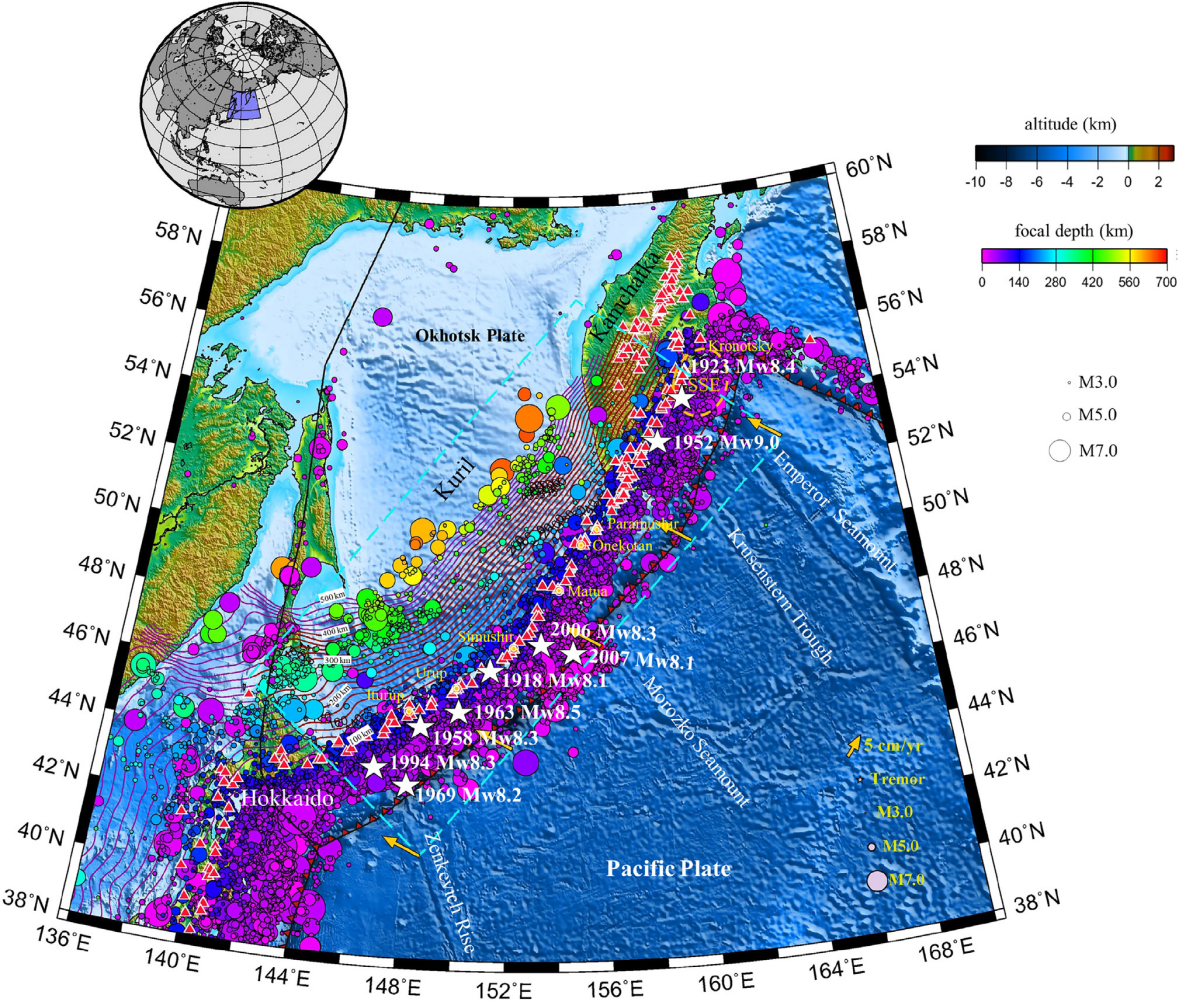
Tectonic map of the Kuril-Kamchatka subduction zone The background color indicates the surface topography (ETOPO^23^). The cyan dashed rectangle indicates the simulated subducting Pacific plate model area. The yellow arrows illustrate that the Pacific plate is subducting beneath the Okhotsk plate in the west-northwest direction. The black lines with red barbs mark convergent plate boundaries. The purple curves represent the isodepth contours on the upper surface of the Pacific plate, with a contour interval of 20 km (Slab 2^24^). Red triangles indicate active arc volcanoes.^25^ The colored spheres indicate earthquakes from January 2000 to December 2010 (IRIS^26^) and M > 5.5 earthquakes from January 1900 to December 2000 (Centennial^27^). The earthquake magnitudes are indicated by size, and the sphere colors imply the focal depth. White stars indicate M > 8 earthquakes.

The circum-Pacific plate belt is traditionally considered to host cold subduction zones, as suggested in cold 2D benchmark models.^1^^,^^12^ To evaluate this endmember thermal model against additional data such as those discussed above, we choose the Kuril-Kamchatka subduction zone as our focus region. As one of the most vulnerable regions in the volcanically active circum-Pacific region, Kuril-Kamchatka is where the Pacific plate is subducting beneath the Okhotsk microplate^13^ (Figure 1). There are 105 subaerial volcanoes and 93 submarine volcanoes in Kuril-Kamchatka,^14^ making the region one of the richest geothermal reservoirs of the circum-Pacific zone.^15^^,^^16^ Kuril-Kamchatka is also among a handful of subduction zones that have experienced giant M ≥ 9 earthquakes in the past century. The Mw 9.0 Kamchatka earthquake on 4 November 1952 caused a catastrophic tsunami with runup wave heights up to 12 m and devastating damage.^17^^,^^18^^,^^19^ M > 8 events are reported to occur on average once every decade within this subduction zone from both instrumental and historical records^20^ (Figures 1, S1B, and S1C). Subslab low-velocity anomalies beneath Kamchatka are revealed by high-resolution seismic velocity tomography, which reflects the potential of trapped/entrained hot material^21^ as an important factor for giant earthquake nucleation and rupture extent.^22^

Generally, slow earthquakes are generated along warm subduction megathrusts such as those below Cascadia, Mexico, and SW Japan because warm overpressured oceanic crust results in a low effective stress on the megathrust, which is a necessary condition for the occurrence of slow earthquakes,^28^ while in cold slabs such as in Kuril-Kamchatka, slow earthquakes should be inhibited. However, slow-slip events in Kuril-Kamchatka were also detected by continuous GPS (Global Positioning System) networks,^29^ which implies that the slab temperature may be warmer than previously thought, as implied by the exhumed rock P-T condition. The estimated slip rates in several areas are higher than the relative velocity of two converging plates, probably indicating fluid overpressure.^30^ Coseismic slip has been suggested to be followed by a slow-slip event with comparable orientation.^31^ These factors necessitate the reestimation of slab thermal states beneath cold plate convergent zones.

The slow and fast earthquakes, as well as ongoing arc magmatism in Kuril-Kamchatka, indicate that slab dehydration, fault weakening, and fluid-facilitated mantle melting are of great significance to this volcanically and seismically vulnerable convergent zone.^14^^,^^32^^,^^33^^,^^34^^,^^35^^,^^36^^,^^37^^,^^38^ Previous cold subduction zone models below Kuril-Kamchatka were based on limited observations involving the surface heat flow near Barkhatnaya Sopka volcano and adjacent earthquakes. Correspondingly, the first main dehydration front downdip of the cold subducting slab was suggested to be associated with eclogitization at subarc or back-arc interplate depths.^39^^,^^40^ However, in warm subduction zones, eclogitization of the oceanic crust provides a source of fluids for the seismogenic zone below the forearc.^41^^,^^42^^,^^43^ Therefore, the mechanism for the intraslab dehydration front inside the downgoing plate of cold subduction zones remains poorly understood, which is ultimately attributable to an inappropriate understanding of slab temperature. For these reasons, this study attempts to revisit the 3D subduction zone hydrothermal structure and intraslab dehydration fronts that vary along the arc to reevaluate the geotherm of subducted plates in Kuril-Kamchatka. We utilize model settings from previous 3D studies that successfully reconstructed cold-to-warm oceanic-continental or oceanic-oceanic subduction systems,^43^^,^^44^^,^^45^^,^^46^ Cascadia,^42^ Hikurangi,^47^ Chile,^48^ Sumatra,^49^ Izu-Bonin,^50^ and Peru.^51^

## Results

### 3D thermal regime of the incoming Pacific plate

**Figure 2:**
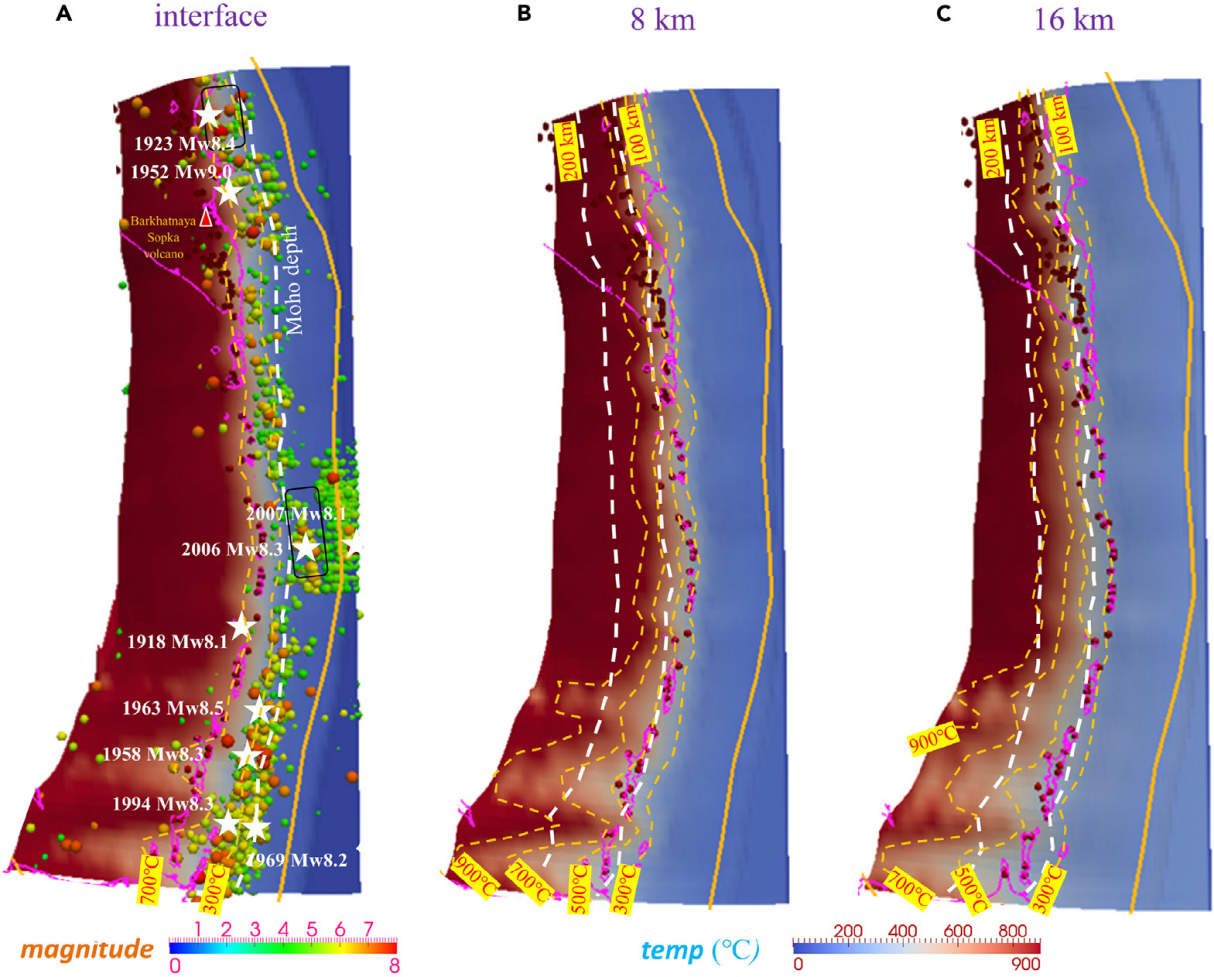
Calculated thermal states at the surface of the incoming plate and various depths from the plate interface (measured perpendicular to the interface) The plotted seismic events with different sphere colors indicate the earthquake magnitude. Red cones indicate active arc volcanoes.^25^ (A) The interface. White stars indicate historical M > 8.0 earthquakes. Yellow dashed curves represent the isotherms. Thin black rectangles with rounded corners indicate the observed afterslips.^53^ (B) 8 km below the interface. (C) 16 km below the interface.

Figure 2 shows the calculated thermal regime on internal surfaces of the incoming oceanic lithosphere with different vertical distances below the plate interface, i.e., 0, 8, and 16 km. The slab temperature decreases at greater depths within the Pacific plate until a cold slab core appears 40 km deep. For example, the subarc interplate temperature is ∼500°C–700°C (Figure 2A), but at a depth of 16 km within the slab, the subarc interplate temperature is ∼300°C–500°C (Figure 2C), which is assumed to be accompanied by active dehydration that facilitates mantle melting and magmatism at the plate interface. Deeper inside the slab, the subarc plate is cooler and reaches a minimum temperature of ∼200°C in the slab core (approximately 38 km below the interface). Therefore, the thermal structure of the subducting Pacific plate is multilayered, which constitutes an important feature of the subduction thermal regime, especially when discussing the deep portion inside the slab. Based on the relatively uniform lithospheric thickness along the subduction zone,^52^ the along-arc thermal regime (300°C and 700°C isotherms) hardly varies from Kronotsky (Figure 1) to the Iturup Islands. However, southward of the Iturup Islands (southern Kurils), the slab geometry remarkably changes from steep subduction to shallow subduction, as illustrated in Figure 2, which significantly changes the slab thermal structure. We calculate the interface temperature and find that the southern Kurils are lower by approximately 100°C than the central and northern Kurils at the same depth, such as 30-km and 50-km depths (Figures S3A–S3D). The distance between the southern Kurils volcanic arc (southernmost red circles indicating volcanoes) and the Moho-depth interface line (southernmost dashed white curve) (approximately 100∼150 km) is markedly larger than that in central and northern Kurils (approximately 80∼100 km) due to the decrease in slab dip angle. The temperature beneath the volcanic arc of the South Kuril Islands is approximately 700°C, while the temperature of the Kamchatka Peninsula exceeds 900°C. In addition, we compared our thermal results with the previous 2D model results^1^ and found that our resulting slab surface temperature is 200°C–300°C higher than the results in previous 2D benchmark models. The concordance of our results with the P-T conditions of the cold endmember of globally exhumed rocks further validates the reliability of our 3D model (Figures S2J and S3E–S3H). In addition, the slab surface in previous benchmark models suggested P-T condition variations near or even inside the forbidden zone (Figure S2J), which is unrealistic even if considering that Kuril-Kamchatka is one of the coldest subduction zones.

### 3D water content variation along the strike

**Figure 3:**
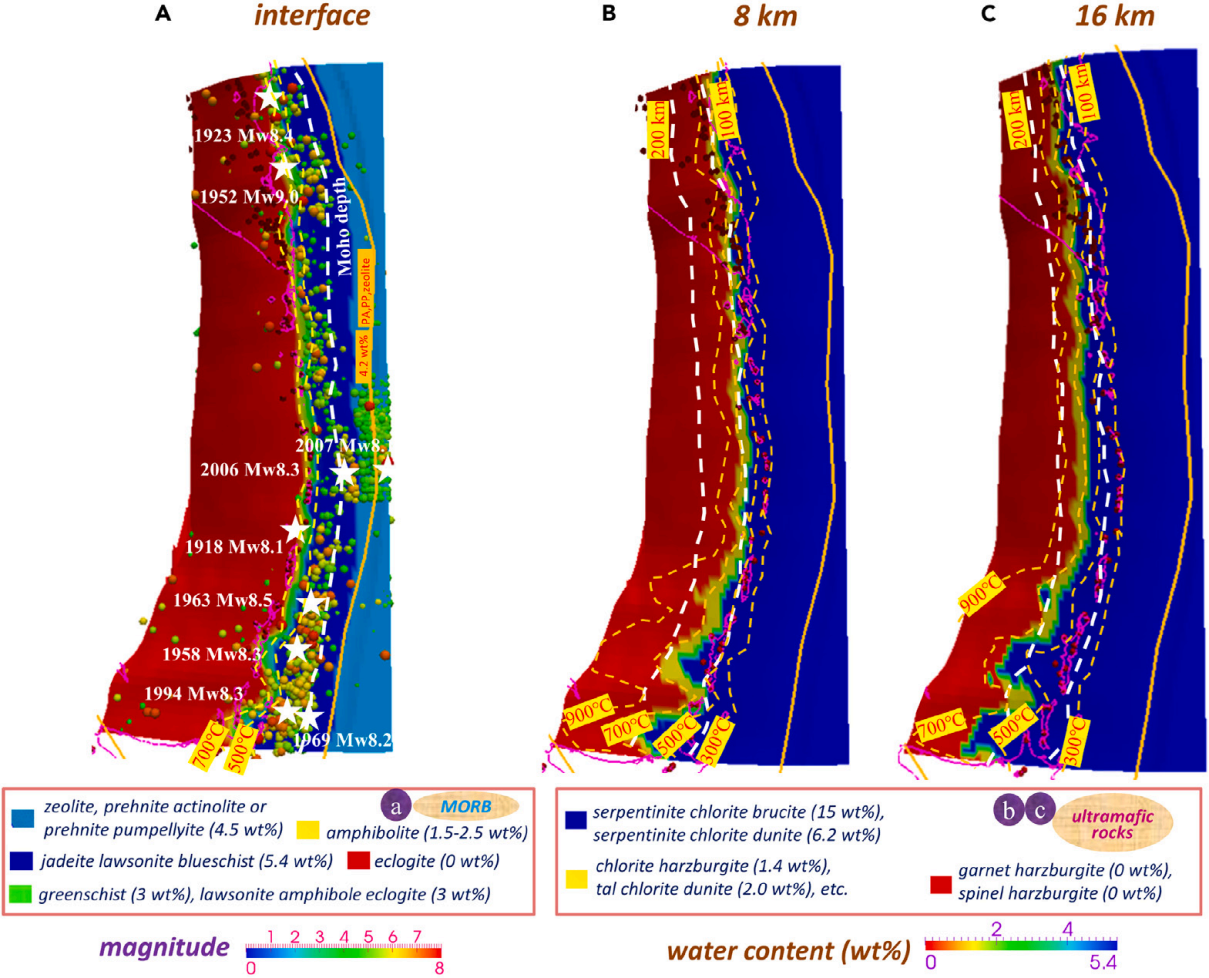
Water content (wt %) of the incoming plate (A) the interface (MORB). (B) 8 km below the interface (ultramafic rocks). (C) 16 km below the interface (ultramafic rocks).

Based on facies diagrams for mid-ocean-ridge basalts (MORBs)^54^ and harzburgite ultramafic rocks^39^ in the subducting circum-Pacific plates, we calculate the three-dimensional slab water content variation at Kuril-Kamchatka (Figures 3, S4A, and S4B). The composition of the upper mantle beneath the northern Kurils^14^^,^^55^ suggests that there is a heterogeneous subduction regime along the Kuril trench between the central and southern Kurils, which is associated with variations in the hydrous state of the topographically curved incoming plate.

**Figure 4:**
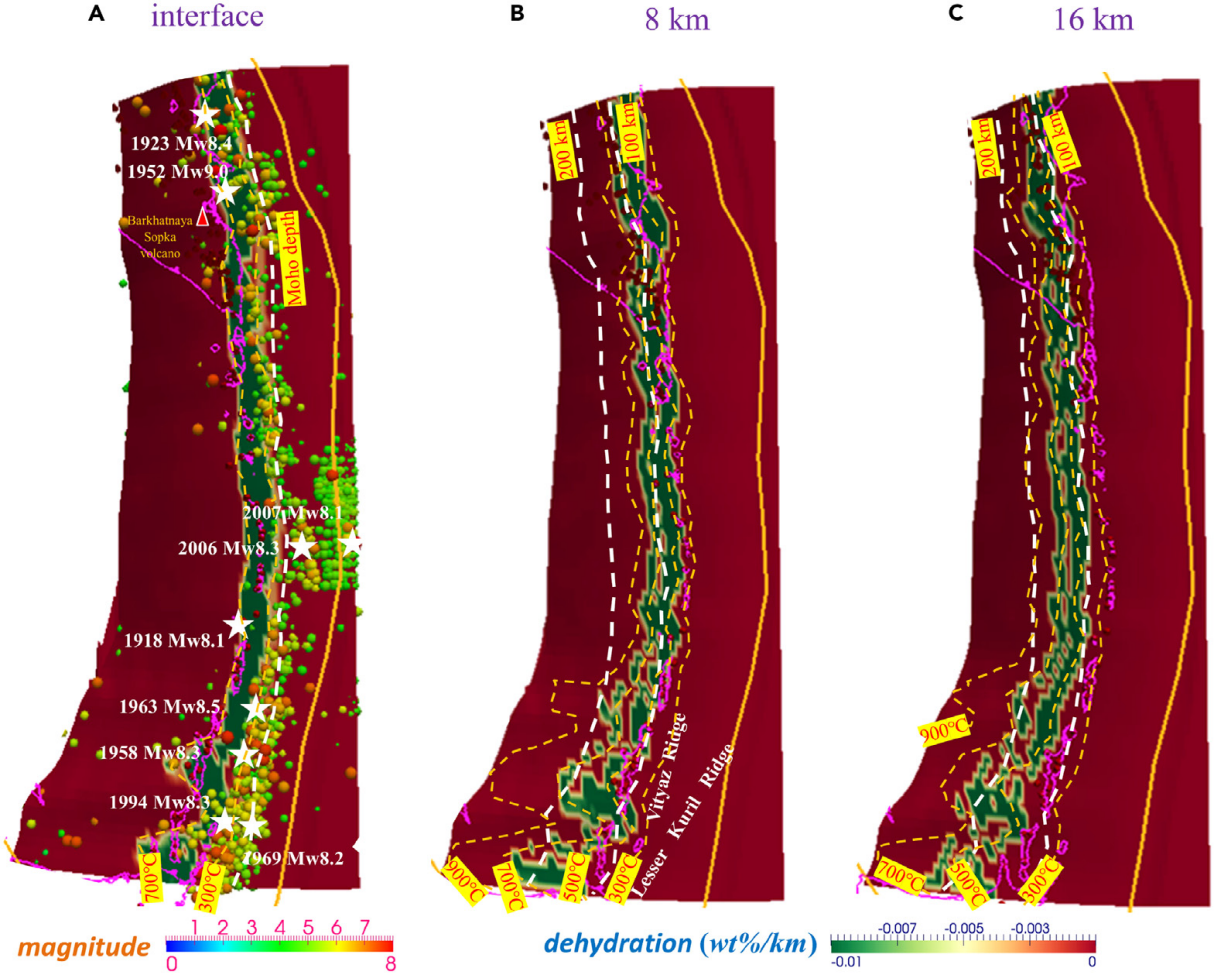
Slab dehydration (wt %/km) of the incoming plate (A) The interface (MORB). White stars indicate M > 8.0 earthquakes. (B) 8 km below the interface (ultramafic rocks). (C) 16 km below the interface (ultramafic rocks).

The water content and slab dehydration distribution on intraslab surfaces with vertical distances from the plate interface of 0, 8, and 16 km are shown in Figures 3A–3C, respectively. Ultramafic rocks are treated as representing the subcrustal (>7 km) oceanic mantle of the incoming plate, and MORB composes the uppermost part of the oceanic plate. On the plate interface, lawsonite blueschist with a water content of 5.4 wt % transforms to lawsonite-amphibole eclogite (3 wt %) at temperatures of approximately 500°C–600°C, followed by eclogite (0 wt %∼) at a temperature of approximately 700°C^39^ (Figure 3A). The change in intraslab water content with temperature increases in ultramafic rock layers at depths of 8 and 16 km. The prediction from the models shows that large amounts of fluids are released at the phase transitions from brucite (15 wt %) to dunite (6.2 wt %) to garnet harzburgite (0 wt %) (Figures 3B, 3C, S4A, and S4B).^39^ We utilized a water content of 15 wt % for the lower subducting oceanic plate because harzburgite is the dominant rock type in the uppermost oceanic mantle, which is largely serpentine at lower temperatures.^39^ However, the estimates remain uncertain because serpentinites at low temperatures (50–300°C) are suggested to be mainly composed of lizardite and magnetite with brucite, but chlorite is not common.^56^ At high pressure, the dehydration of serpentinite forms antigorite serpentinite, chlorite harzburgite, and then garnet peridotite.^56^^,^^57^ The degree of serpentinization in the mantle wedge based on seismic velocities ranges widely and has a certain uncertainty.^58^ For further analysis, we converted the water content to slab dehydration, which reflects the efficiency of fluid production during slab subduction compared to the tectonic background. Remarkably, beneath the Kuril-Kamchatka volcanic arc, the >1,700-km-long slab dehydration sliver on the interface (Figures 4 and S4C–S4F) shows a dehydration efficiency of >0.01 wt %/km, which is obviously larger than that on other portions of the interface, such as the segmented updip and downdip patches. At 8 km below the megathrust, dehydration fronts migrate westward of the arc and downdip of the subarc sliver. Beneath the southern Kurils, the dehydration fronts migrate significantly westward of the arc compared with those under the central and northern Kurils due to flattening slab subduction. As inferred from the models, the variation in slab geometry along strike markedly changes the dehydration fronts at depths beneath the southern Kurils and causes possible slab segmentation due to high slab curvatures (Figures 4B and 4C).

## Discussion

### 3D thermal regime and slow and fast earthquakes

To investigate the thermal control and its potential correlation with seismicity, we incorporate new surface heat flow measurements and Curie depths to constrain our slab-mantle model to fit the observations of seismicity, volcanism, and surface heat flow (Figures 1, S1, and S2). The interface temperature nearest to the mainshock sources with magnitudes M > 8 is approximately <500°C in the south-central Kurils but 500°C–700°C below Kamchatka (Figure 2). In addition, the progressively shallower Pacific plate southward is associated with colder subduction conditions (approximately 300°C–500°C), so M > 8 earthquakes occur more frequently in the southern Kurils (Figure 2A). In parallel, we compared the 3D thermal structure model with the benchmark models^1^^,^^59^; notably, the forearc interplate temperature calculated by Wada and Wang^59^ is colder by 200∼300°C than our calculation because their slab surface temperature is greatly depressed by 300°C∼ due to the prescribed maximum decoupling depth,^59^ and our result without such plate decoupling indicates a higher interface temperature than that of the plate decoupling models beneath the forearc and exhibits a smooth interplate temperature transition beneath the arc (Figures S2J and S3E–S3H). Cerpa et al.^60^ proposed that the geotherms of South Kuril near 6 GPa and 580°C were based on the thermal structure with a coupling depth of 65 km. Considering the choke point pressure (5.5 GPa), our modeled thermal results suggest an elevation of ∼300 °C at the slab surface and a large increase in the water budget released above this choke point. The temperature conditions above 3 GPa (Figures S2J and S3A–S3D) approximate the results of previous studies and are supported by the P-T conditions of globally exhumed rocks.^11^^,^^61^

According to the statistics of the relationship between earthquake numbers and depth from 1 January 1900 to December 2010, we noticed that giant (M > 8) earthquakes were closely distributed updip of the seismogenic interfaces at depths near the Moho (20–40 km) (Figure S2K). For regular interplate earthquakes, most of the recorded events are mainly clustered in a thermal state of 300°C–700°C within a sliver zone on the slab surface in our model, which is predicted slightly eastward of the Kuril-Kamchatka volcanic arc, i.e., approaching the Moho-depth megathrust (Figure 2A). The calculated downdip thermal gradient on the upper surface indicates a high value of >5 °C/km from Kamchatka to Urup Island (Figures S5A–S5C). This gradient (∼5 °C/km) is largely caused by the increasing slab dip angle, which forms a marked slab curvature at subarc depths (Figures S5A–S5C). Lawsonite was identified to carry water to great depths (as large as 300 km) in the cold subduction zone with a thermal gradient <5 °C/km.^62^ However, the high downdip thermal gradient in the cold subduction zone is expected to result in a rapid phase transformation within the intraslab hydrous minerals and contribute to immediate slab dehydration.^47^ Therefore, regular interplate earthquakes and tectonic tremors occur on or adjacent to the plate interface with >5 °C/km for the thermal gradient.

Since the thermal structure of the subducted Pacific plate is hierarchical and heterogeneous with changing depth, we focused on the changes in thermal structure inside the slab and along the strike, which affect the distribution of fast and slow earthquakes. In the northern part of our calculated model, a thermal change appears near Kronotsky (eastern coastal Kamchatka Peninsula, approximately 54°N). The mechanism is unclear and possibly attributed to the northwestern extension of the Emperor Seamount chain, which accounts for the increase in plate dip angle and nearby seismicity.^63^^,^^64^ North of 54°N, the dip angle below Kamchatka decreases to approximately 35°. The subducted Morozko Seamount influences the seismicity patterns and thermal structure, which reflects a low-temperature phenomenon (low surface heat flow) and an afterslip area that is impacted by this trough (Figure 2A). A high probability of long-term SSEs (slow slip events) in the central portion of Kuril-Kamchatka is also suggested based on a limited number of GNSS (Global Navigation Satellite System) instruments.^65^ Calculation suggests that the *in situ* temperatures in the fault zones are lower than those in other nearby areas, and the plate contact becomes more fragile and prone to slip, causing trenchward clustered earthquakes and afterslips in the central Kuril-Kamchatka trench (Figures 2A, 3A, and 4A). The subducted MORB layer is calculated to be approximately 100∼200°C cooler than the interplate sediments at depth, which facilitates the flushing of subducted sediments, and the dehydrated MORB layers provide the required amount of fluid through amphibole and chlorite decomposition reactions.^66^ Bürgmann et al.^29^ suggested that seismic deformation was controlled by temperature, which transitioned to aseismic slip at 300–400°C and viscous flow at higher temperatures. This interpretation is supported by our study: the two aforementioned slow-slip zones are distributed at approximately 300∼400°C (Figure 2A).

### Slab dehydration front variation and slow and fast earthquakes

**Figure 5:**
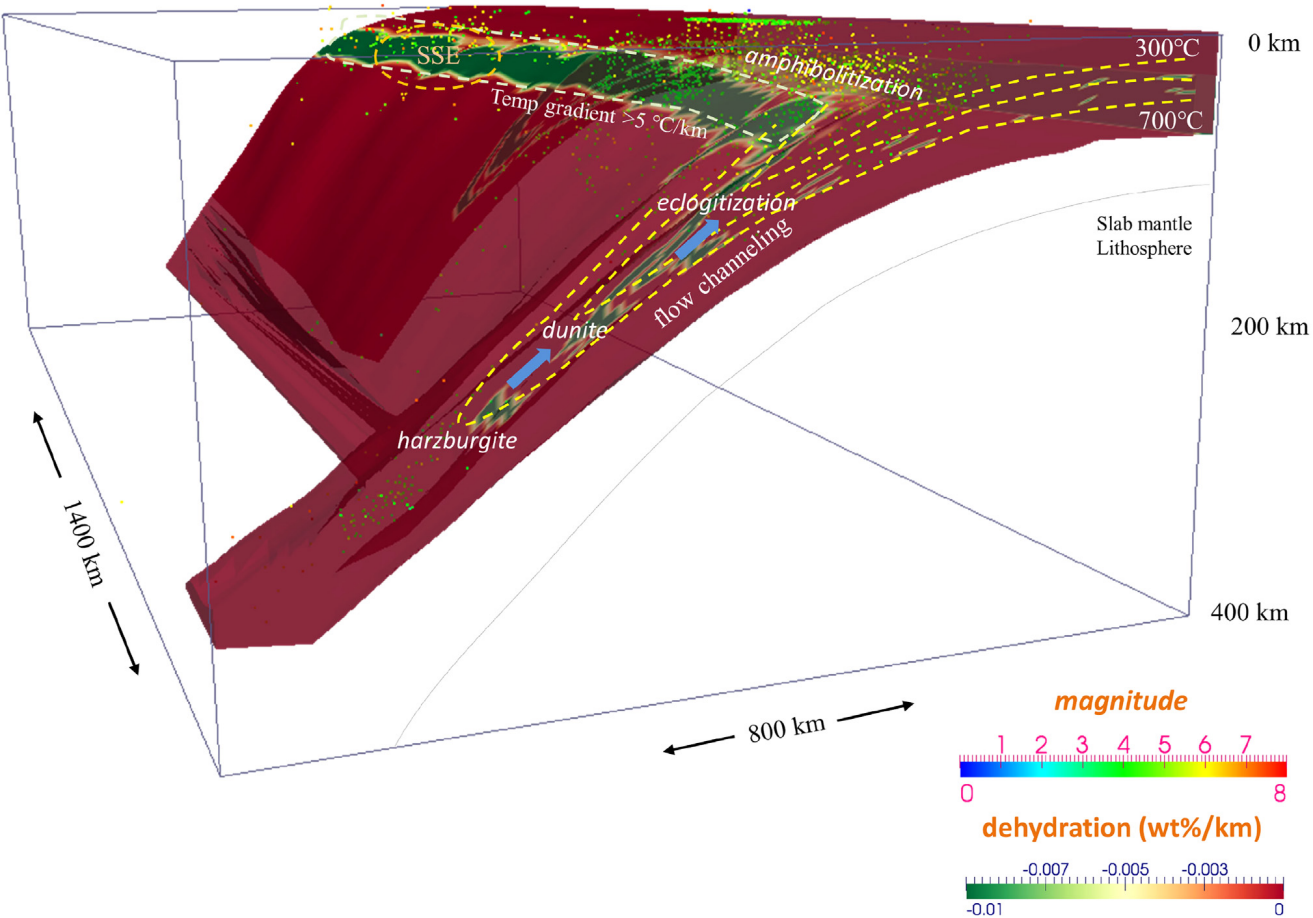
Multilayered dehydration fronts are shown in the subducted oceanic plate, including rock amphibolitization, eclogitization, and the phase transition to dunite and harzburgite beneath Kuril-Kamchatka The limit of the cold nose (<500°C) is restricted to the trenchward part of the mantle wedge. The slab surface colored green indicates a temperature gradient of >5°C/km along the slab surface, where deep SSE usually occurs. Fluids are transported from the dehydration fronts to the shallow portion of the slab via flow channeling and contribute to mantle melting and slab weakening.^5^

Via calculation, we find that the shallow seismogenic zone is distinguishable and associated with the high water content transition boundaries, i.e., slab dehydration fronts (Figures 4 and 5). The water content distribution shows a notable sliver zone transverse from Kamchatka to Kuril on the interplate surface. The light blue coloration indicates the prehnite-actinolite, prehnite-pumpellyite, and zeolite assemblages with a water content of nearly 5 wt % distributed near the plate margin, where comparatively few earthquakes occur (Figure 3). Massive seismic events occur adjacent to the amphibolitization and eclogitization fronts, and shallow earthquakes are located at the transitions from lawsonite blueschist (5.4 wt %) to amphibolite (1.5–3 wt %) to eclogite (0 wt %∼) (Figure 3A). Underlying the Okhotsk plate and deeper portion of the coupled subduction interface (<250 km), the earthquake distribution coincides with the calculated dehydration strip. When the depth of the subducted plate increases, the remarkable influence of dehydration is caused by changes in rock facies from serpentinite-chlorite brucite (15 wt %) to serpentinite-chlorite dunite (6.2 wt %) in these seismic zones (Figure 3C). A deep dehydration area is produced by ultramafic rocks, which release a majority (>75%) of the fluids and induce significant upwelling of fluid into the continental wedge. The presence of this deep hydration area corresponds with the fronts of phase transition to dunite and harzburgite within the incoming cold oceanic plate (Figures 4, 5, S4A, and S4B).

Medium-large earthquakes (M > 5.5) are mostly located 20–50 km beneath the forearc region, which is near the interface dehydration fronts (Figure 4). Since great earthquakes may be facilitated by low temperatures and slab dehydration of >0.05 wt %/km for foreshocks,^48^ the rupture of M > 8 earthquake sources beneath Kuril-Kamchatka may also be facilitated by MORB amphibolitization and eclogitization. The constancy between slab dehydration fronts and recorded large-medium earthquakes is noticeable on the subduction megathrust beneath Kuril-Kamchatka (Figure 5). Coseismic and postseismic slips are also identified in the source regions of the 1997 and 2004 M > 8 earthquakes based on GPS station velocity variations.^31^^,^^53^ Wada et al.^12^ predicted the dehydration of the subducting crust peaks at depths shallower than 120 km in the cold-slab model. Konrad-Schmolke et al.^63^ suggested a dehydration pattern with a significant increase in the amount of water release at approximately 120 km beneath the Kamchatka Peninsula. In our model, the structure of dehydration fronts is stratified and imposes a characteristic effect on fault instability and serpentinite breakdown from shallow portions to depths that reach an intraslab core complex depth of >150 km (e.g., Figures S4C–S4F and S5D).

In this study, regular interplate earthquakes and tectonic tremors occur with high slab dehydration efficiency and high downdip thermal gradients. Abundant arc magmatism occurs above the uppermost stratified intraslab dehydration fronts, including the phase transition to dunite and harzburgite. Most of the intermediate-depth seismicity (∼140 km) that occurs in the subducting plate beneath Kuril-Kamchatka is potentially connected to intraslab multilayered dehydration fronts (Figure 5). Intraslab fractures that experience intense normal faulting during slab bending develop at depth and greatly contribute to slab segmentation, viscous weakening, and fluid channeling.^5^ Furthermore, since the subducting plate changes curvature in three dimensions, the stress field rotates, and the fluid pore pressure can be affected and follow areas where the surrounding stress allows them to flow, which finally causes seismicity via fluid-related embrittlement.^67^^,^^68^ In this regard, slab bending and continuously strengthened intraslab normal faulting can affect flow channeling within dehydration front networks and cause subarc and subforearc large-scale fluid/melt confluence and migration.

### Conclusions

Using a 3D thermal model of the downgoing oceanic lithosphere beneath Kuril-Kamchatka, for the first time, we reestimated the slab temperature and dehydration based on the observed P-T conditions of exhumed rocks and obtained the following results.

(1) The medium-high forearc surface heat flow and global exhumed rock P-T conditions indicated that the subducted slab is much warmer than that predicted by previous 2D thermal models by 200∼300°C, while our new 3D reestimation of the thermal state of the Kuril-Kamchatkan slab supports the observations involving exhumed rock records.
(2) The hierarchical slab dehydration fronts vary in a complicated manner along the strike and are effectively described via 3D thermal modeling for the first time.
(3) The multilayered subduction regime and a large downdip thermal gradient of >5 °C/km beneath Kuril-Kamchatka indicate a stratified characteristic effect on slab dehydration efficiency.
(4) The frequent recurrence of fast-to-slow subduction earthquakes in Kuril-Kamchatka is facilitated by multiple thermally controlled petrological metamorphic processes that evolve with the slabs.

### Limitations of the study

Both active slow and fast earthquakes and arc magmatism in Kuril-Kamchatka indicate that hydrothermally controlled slab dehydration, fault weakening, and fluid-facilitated mantle melting are of great significance. In this study, we focus on the 3D subduction hydrothermal structure varying with depth. Current limitations of the study are some uncertainties of the hydration of oceanic mantle. We utilized a water content of 15 wt % for the lower subducting oceanic plate because harzburgite is the dominant rock type in the uppermost oceanic mantle, which is largely serpentine at lower temperatures.^39^ However, the estimates remain uncertain because serpentinites at low temperatures (50–300°C) are suggested to be mainly composed of lizardite and magnetite with brucite, but chlorite is not common.^56^ At high pressure, the dehydration of serpentinite forms antigorite serpentinite, chlorite harzburgite, and then garnet peridotite.^56^^,^^57^ The degree of serpentinization in the mantle wedge based on seismic velocities ranges widely and has a certain uncertainty. We recognize that the calculated maximum water content may decrease from 15 wt % to 2–3 wt % if the assumption is made that serpentinite is the only water-bearing and dehydrated rock type in the subducted lithospheric mantle before the choke point (6 GPa and 580°C).^60^

## STAR★Methods

### Key resources table

**Table:** 

REAGENT or RESOURCE	SOURCE	IDENTIFIER
**Deposited data**		

3-D model results in this study	Mendeley Data	https://data.mendeley.com/datasets/jgy68s9ttg
Stag3D code	Tackley et al.^70^	https://doi.org/10.1016/B978-008044046-0.50372-9
Incorporated Research Institutions for Seismology	Trabant et al.^26^	https://doi.org/10.1785/0220120032
Slab 2	Hayes et al.^24^	https://doi.org/10.1126/science.aat4723
ETOPO Global Relief Model	Smith et al.^23^	https://doi.org/10.1126/science.277.5334.1956

**Software and algorithms**		

Generic Mapping Tools	Wessel et al.^69^	https://doi.org/10.1029/98EO00426
Paraview	Kitware Inc.	https://www.paraview.org/license/

### Resource availability

#### Lead contact

Further information and requests for resources should be directed to the lead contact, Yingfeng Ji, yingfengji@itpcas.ac.cn.

#### Materials availability

This study did not generate new unique reagents.

### Experimental model and subject details

Our study does not use experimental models.

### Method details

To explore slab thermal and hydrous states, we construct a 3-D, time-evolving thermomechanical finite difference model based on the Stag3D code.^70^ The model dimensions are 1800 km × 800 km × 400 km, which indicate that x = 1800 km along the strike of convergence, y = 800 km in the cross-arc direction, and z = 400 km in the vertical direction (Figure S1C). In this study, we use the equations of conservation of mass, momentum, and energy.^46^^,^^71^ We obtain age data for the incoming plate following the oceanic seafloor age, which is estimated by the trenchward model boundary based on EarthByte.^72^ As an old and cold Early Cretaceous oceanic crust, the seafloor age varies from 102 to 106 Myr at the Kamchatka Peninsula to 112 Myr at Simushir Island and 126 Myr near Hokkaido (Figure S1B). The slab thickness is estimated according to the plate age.^73^ The temperature boundary condition is consistent with the plate cooling model.^74^ The bottom of the slab and perpendicular plane are prescribed as adiabatic and permeable, and the top surface is set to be rigid with a fixed temperature (0°C) (Figure S1C). The 3-D geometric data for the incoming plate adopt updated seismicity-based slab geometry (Slab2;^24^). The intraslab velocity field follows the MORVEL datasets^75^ and slab geometry,^24^ where the x, y, and z velocity components are computed at each grid based on the kinematic modeling method.^45^ The slab convergence velocities vary from 8.3 mm/yr in the southern Kurils to 7.7 mm/yr in the central Kamchatka Peninsula. This convergence is applied at the trench to ensure that the slab subducts to depth following the prescribed slab guide of Slab2 (Figure S1C). The viscous flow law for wet olivine,^76^ which was determined through laboratory experiments, is incorporated into the model use (Tables S1 and S2). The deformation of olivine includes diffusion creep and dislocation creep, which accommodate the total strain rate.^77^ Utilizing the temperature and pressure conditions provided for subducted rocks,^39^^,^^54^ we estimate both the facies domain and corresponding water content (wt %) at every grid inside the slab. We also established a P–T-wt%-facies database according to Omori et al.^54^ (MORB) and Hacker et al.^39^ with a P–T grid interval of nearly 0.04 GPa (1.2 km) and 5°C (Figures S1D and S1E). Via interpolation, we derive the intraslab temperature (°C) and water content distribution (wt %) at different intraslab depths. The slab temperature gradient (°C/km) and dehydration rate (wt %/km) are thus calculated using the intraslab temperature/water content change divided by a distance (km) in the subduction direction between neighboring grids. The modeling results are constrained by surface heat flow observations^78^ and heat flow from the Curie point depth estimates^79^ (Figures S2A–S2I). According to the comparison between thermal models and exhumed rock P–T conditions (Figures S2J), our models exclude the effect of frictional heating following previous thermal modeling,^80^ which considered that shear heating had a limited effect on water circulation.^4^

### Quantification and statistical analysis

We tested the resolution and found that the temperature variance was <1% with a maximum temperature variance <1.9% between meshes of 80 × 80 × 100 and 96 × 96 × 100. We performed sensitivity tests to investigate the robustness of our modeling results and varied the mantle viscosity from 1.0 × 10^19^ Pa s to 1.0 × 10^21^ Pa s and the mantle density from 3250 kg/m^3^ to 3350 kg/m^3^. We present the benchmark model results as deviations from the reference models (ΔT and ΔH_2_O) and show these results at different depth levels within the oceanic slab. The tests show that mantle density variations (±50 kg/m^3^) induce small temperature variations of <10 °C at depth. The benchmark models included the plate-decoupling and frictional heating models, where the results exhibited less correspondence to the surface heat flow observation at the forearc than the result of the reference models using the least square method (Figures S2A–S2I). The cold nose in Kuril-Kamchatka is constrained to a depth of approximately 50∼60 km, which is graphically at the midpoint between the arc and the Moho-depth plate interface (Figure 2A).

## Data Availability

• The data produced in this study can be downloaded (publicly accessible) via Mendeley Data: https://data.mendeley.com/datasets/jgy68s9ttg. • This paper does not report original code. • For any additional information required to reanalyze the data reported in this paper, please contact the lead author.

## Supplemental information

Documen S1. Figures S1–S5 and Tables S1 and S2
