# Supplementary material for: Reestimation of slab dehydration fronts in Kuril-Kamchatka using updated global subduction zone thermal structures
Source: iScience. 2023 Jul 11;26(8):107288. doi: 10.1016/j.isci.2023.107288 (PMC10382886; doi:10.1016/j.isci.2023.107288)
Supplement: Documen S1. Figures S1–S5 and Tables S1 and S2 [file mmc1.pdf]

iScience, Volume *26*

Supplemental information

Reestimation of slab dehydration fronts in Kuril-Kamchatka using updated global subduction zone thermal structures

Weiling Zhu, Yingfeng Ji, Lijun Liu, Rui Qu, Ye Zhu, Chaodi Xie, and Lin Ding

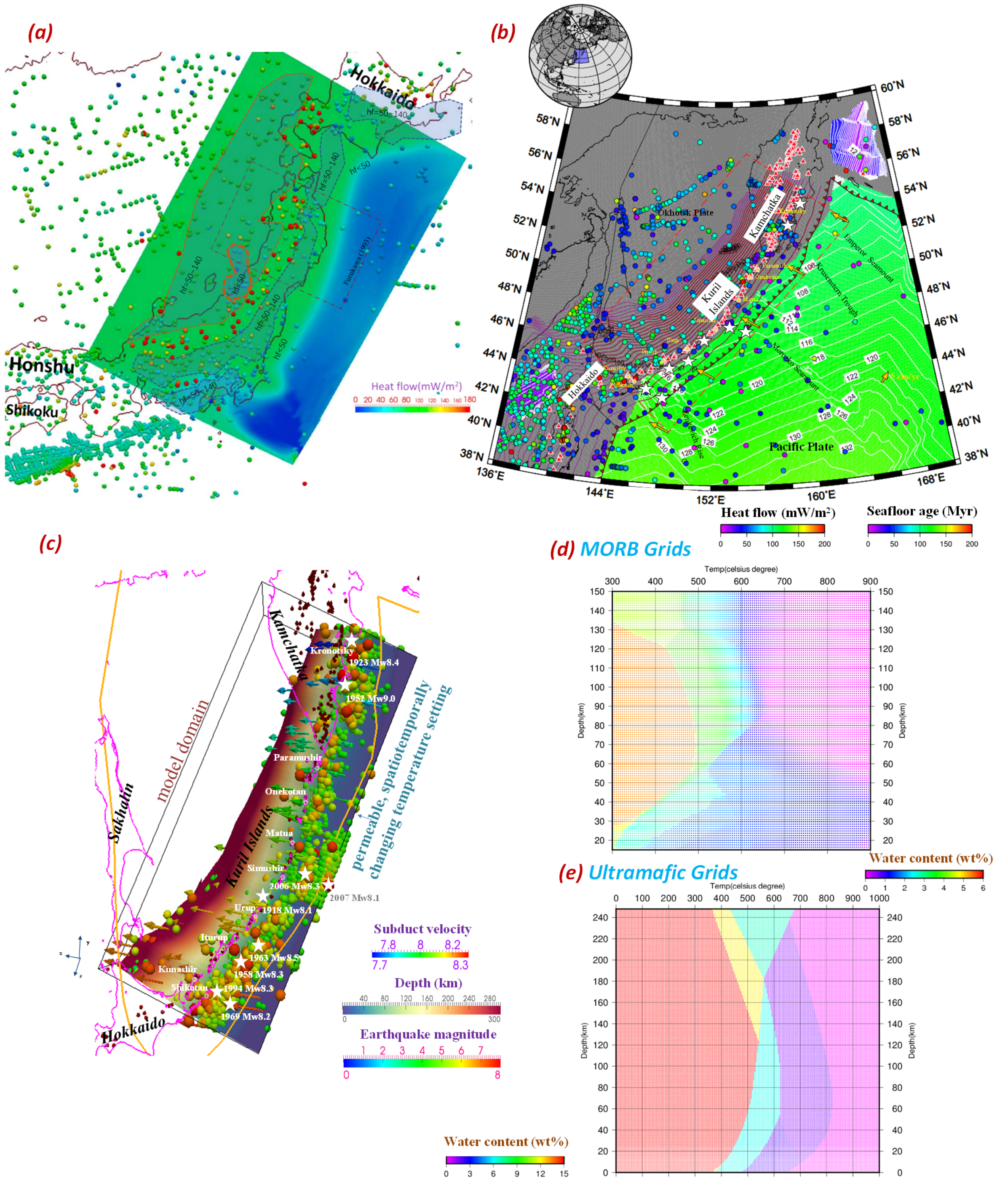

Figure S1. Surface heat flow, seafloor age, model settings, boundary conditions and temperature-depth phase diagram with P-T grids; related to Figure 1 and STAR Methods. (a) The integrated heat flow observations were based on the Global Heat Flow Database [1-7]. We found a forearc medium heat flow region (50–140/150 mW/m2) in northeastern Japan, indicating that plate coupling occurs at a forearc depth of 40–80 km, and the plate decoupling hypothesis is not robustly constrained by heat flow observations. (b) Solid circles represent observations from the Global Heat Flow Database [1]. The seafloor age is from EarthByte [8]. (c) The seismic events are plotted as spheres whose color indicates the earthquake magnitude. Red cones indicate active arc volcanoes [9]. The white stars indicate M>8 earthquakes (focal depth < 100 km) from January 1900 to December 2010 (USGS). (d-e) Temperature-depth phase diagram (inferred from the preliminary reference Earth model) for MORB [10] (d) and ultramafic [11] (e) rocks with all of the P–T grids (colored small solid circles) used for the calculation of slab water content in this study.

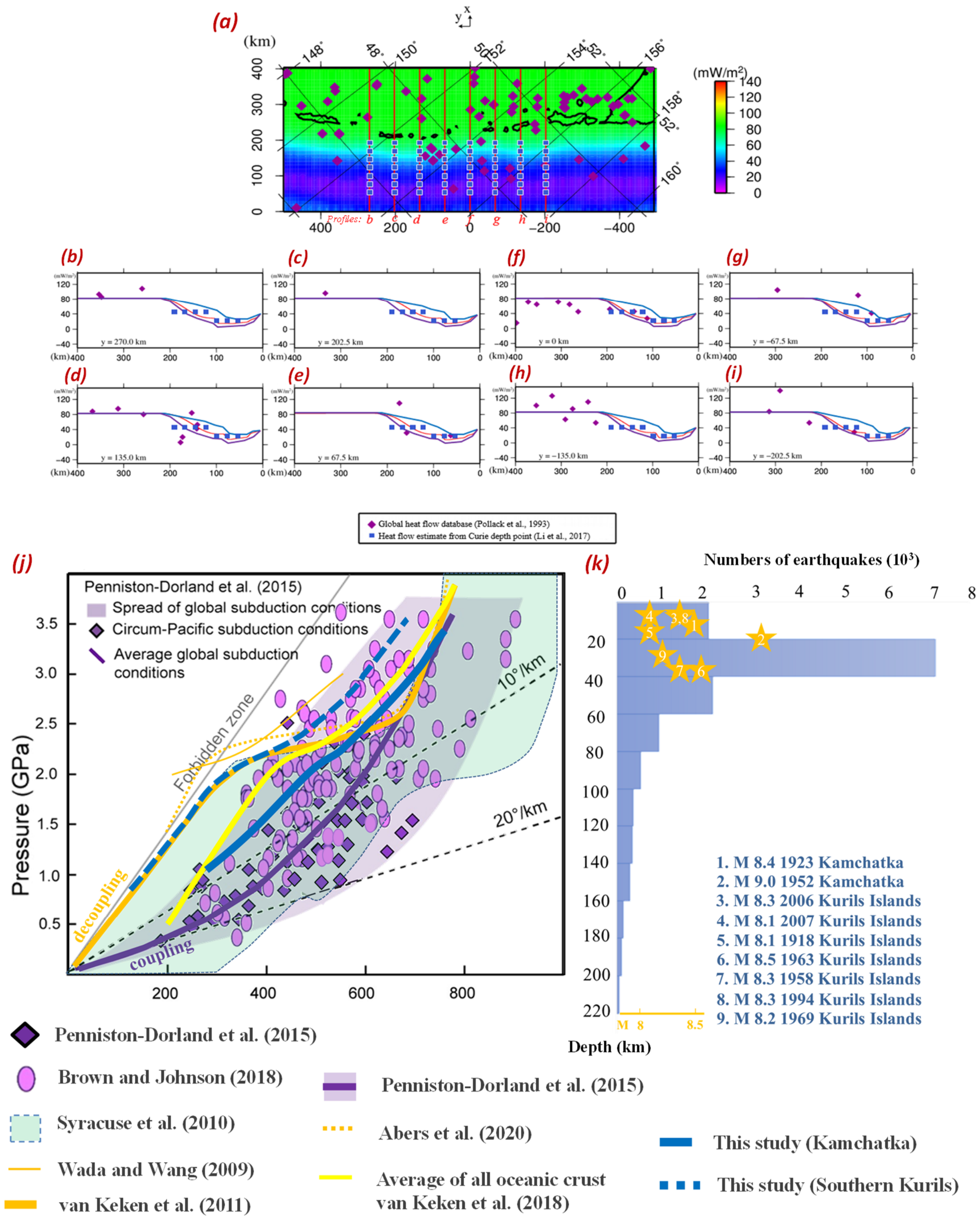

Figure S2. Spatial distribution of the surface heat flow and the comparison of the P–T conditions with previous models; related to STAR Methods and Figure 2. (a-i) The observed and calculated surface heat flows are compared along trench-normal profiles "b" through "i" in panels (b) to (i). The thermal conductivities K are assumed to be 1.5 W/m°C for the ocean [12,13] and 2.5 W/m°C for the continent [13]. Purple diamonds and blue squares denote heat flow data [1] and heat flows from the Curie point depth estimates [13], respectively, within 33.75 km of the profile line. The colored curves indicate the calculated heat flow along the profile. The red curve indicates the calculation without plate decoupling or frictional heating, while the blue curve denotes the calculation with frictional heating (frictional coefficient = 0.05), and the purple curve represents the calculation with plate decoupling (maximum decoupling depth = 100 km). Among them, the red curve fit the observation best. (j) Comparison of the P–T conditions between this study, previous models [14,15], and the global range of subduction zone thermal structures from exhumed blueschists and eclogites [16,17]. (k) Earthquake number-depth histogram for all earthquakes (<220 km) from January 1900 to December 2010 (USGS). The yellow stars indicate the M>8.0 earthquakes.

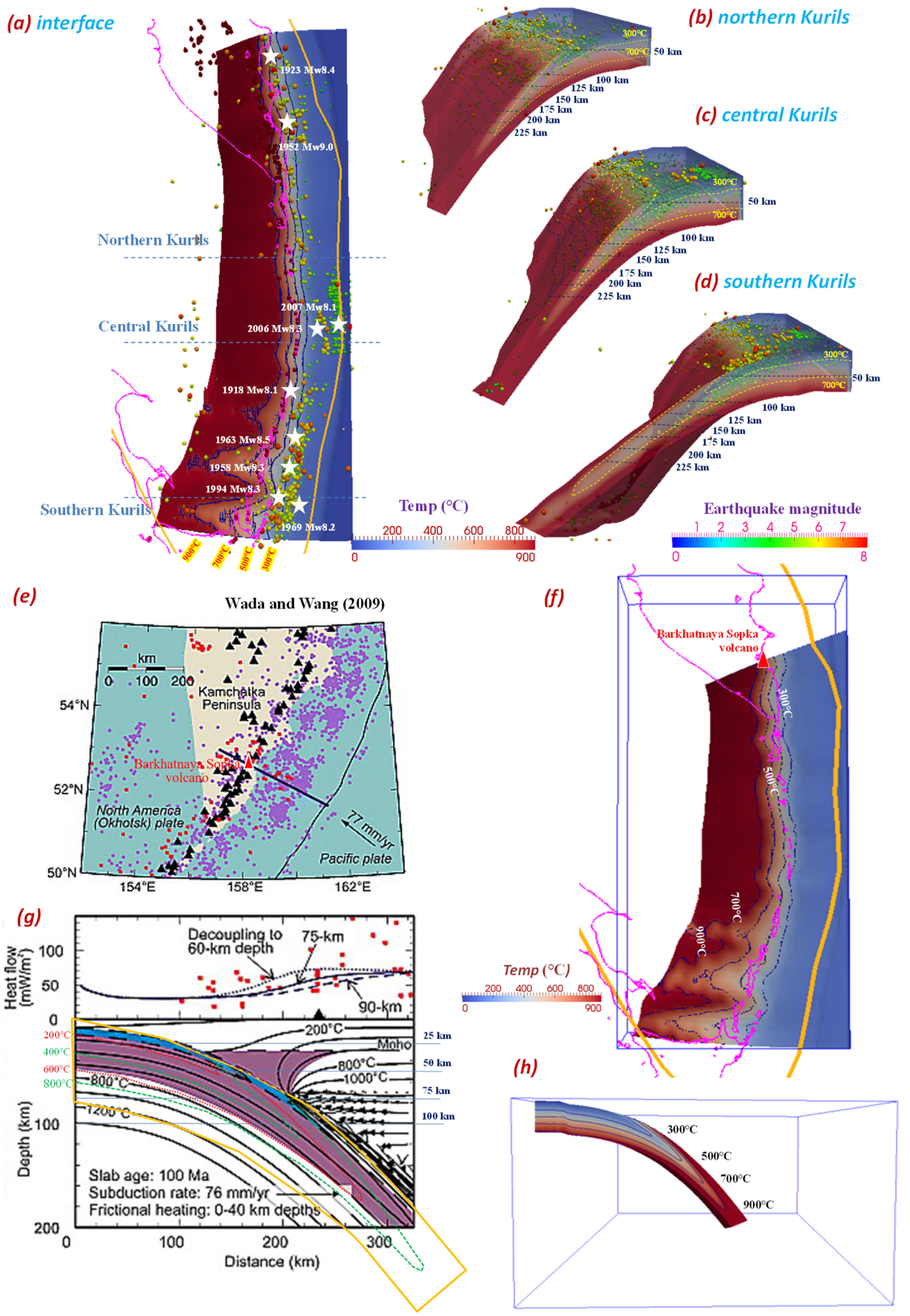

Figure S3. Cross-section of the thermal structure and the comparison of model results between this study and Wada and Wang [15]; related to Figure 2. (a) The colored spheres and white stars indicate the recorded earthquakes. (b)-(d) Cross-section of the thermal distribution along the profiles of the three blue dashed lines in (a). The yellow dashed lines indicate the iso-thermal contours (300°C and 700°C), while the blue dashed lines are the iso-depth contours (50-225 km). (e) Profile used to estimate the cross-section thermal structure in Kamchatka by the 2-D model [15]. (f) Profile obtained from the 3-D model in this study. (g) Difference in slab geometry and calculated thermal structure between Wada and Wang [15] and this study. The black curves and symbols are from Wada and Wang [15], while the yellow curves (slab domain) and red–green curves (intraslab thermal contours) are from this study. (h) Cross-section thermal structure of this study along the same profile as Wada and Wang [15] in a 3-D perspective.

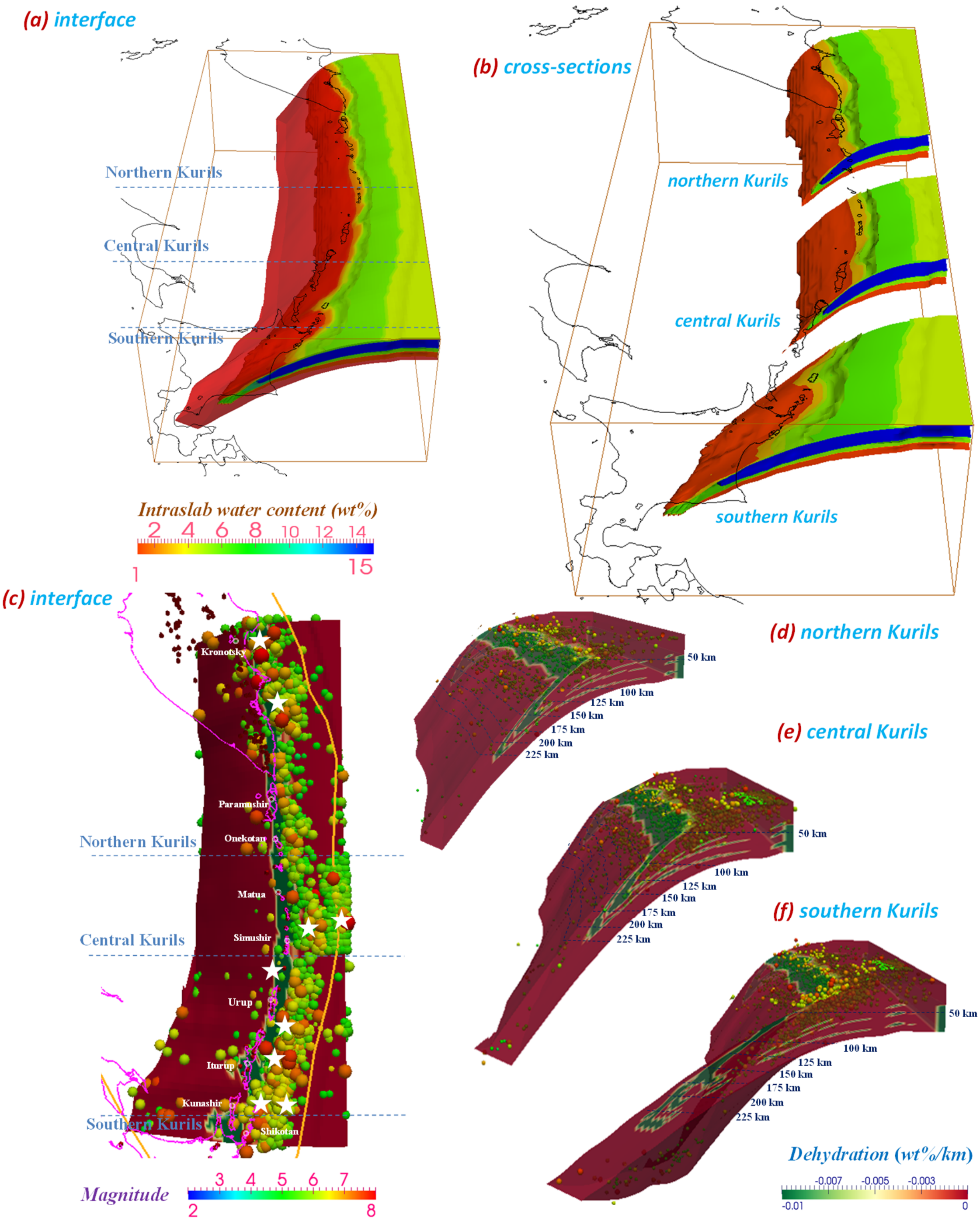

Figure S4. Intraslab water content (wt%) distribution and slab dehydration along the three cross-sections; related to Figures 3 and 4. (a-b) water content (wt%) distribution on the plate interface (a) and along the three cross-sections in the northern, central, and southern Kurils (b). The colors indicate the calculated slab water content of >1 wt% (b). (d)-(f) Cross-sections of the slab dehydration distribution along the three profiles in (c). The dashed blue lines indicate the iso-depth contours (50-225 km). The slab color indicates the slab dehydration gradient (wt%/km). The colored spheres and white stars indicate the recorded earthquakes.

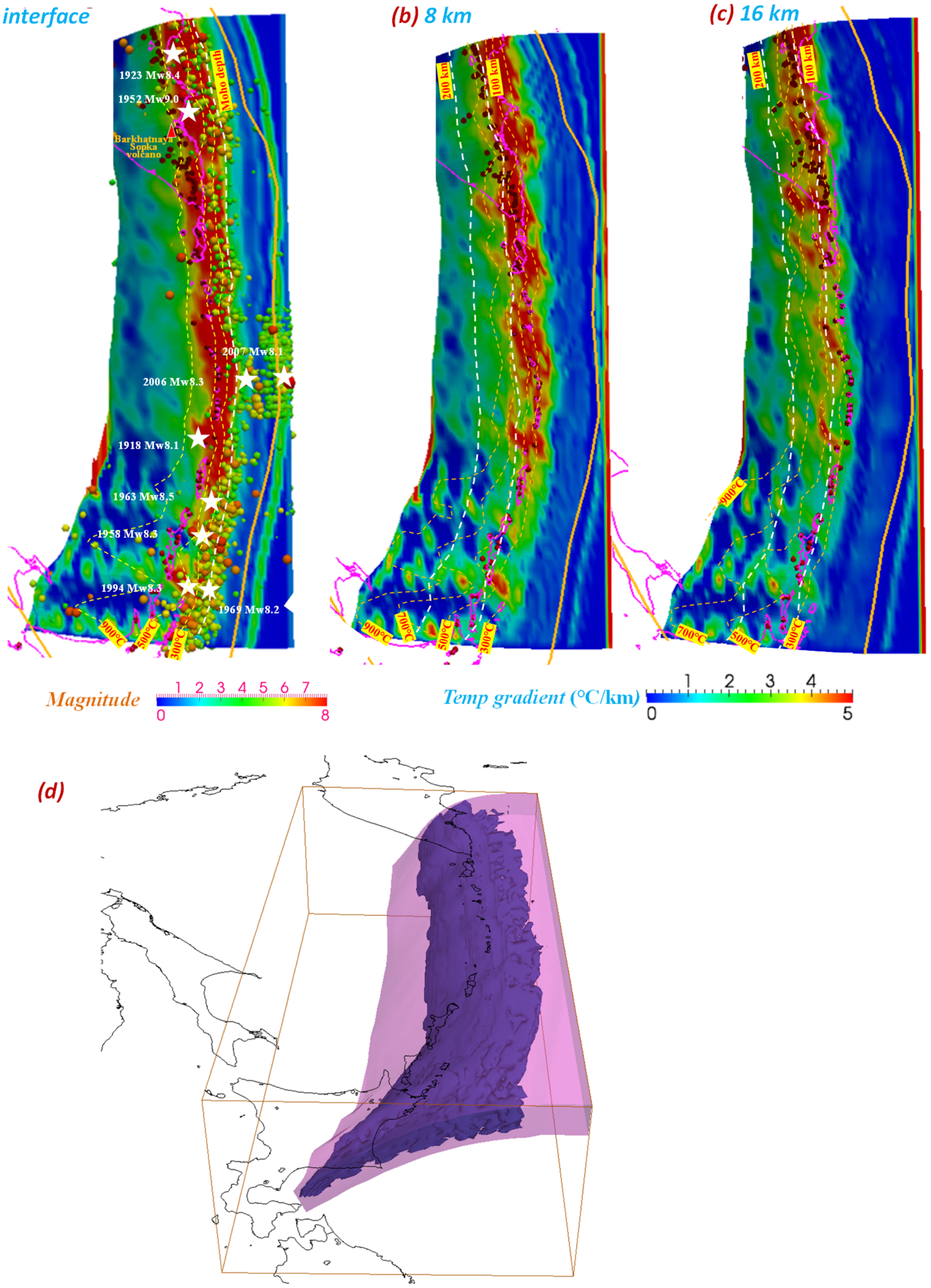

Figure S5. Temperature gradient (°C/km) and multilayered dehydration fronts of the incoming plate; related to Figures 2, 5, and STAR Methods. (a) The interface (MORB). White stars indicate M>8.0 earthquakes. (b) 8 km below the interface (ultramafic rocks). (c) 16 km below the interface (ultramafic rocks). (d) The dark slate blue color indicates the complex of multilayered dehydration fronts combining rock amphibolitization, eclogitization, dunitization, and partial harzburgitization (>1 wt%) within the subducted plate beneath Kuril-Kamchatka. The light slate blue color indicates the domain of the subducted Pacific Plate.

Table S1. Main model parameters; related to STAR Methods.

	Model Parameters	Value	Unit
ρ_0	Standard density	3300 [18]	$kg\cdot m^{-3}$
α_0	Standard thermal expansion	3×10^{-5} [19]	K^{-1}
T_0	Standard temperature	1600	K
k_0	Standard thermal conductivity	2.9 [20]	$W\cdot m^{-1}\cdot K^{-1}$
Hr	Radioactive heat generation rate in the mantle	2.245×10^{-13} [18]	$W\cdot m^{-3}$
C_{p_0}	Standard specific heat at constant pressure	1200 [18]	$J\cdot kg^{-1}\cdot K^{-1}$
κ_0	Standard thermal diffusivity	7.6×10^{-7} [21]	$m^2\cdot s^{-1}$
η_0	Standard viscosity	1×10^{20} [20]	Pa·s
v	Subduction velocity	6.6–7.2 [22]	$cm\cdot y^{-1}$

		Diffusion creep [23]	Dislocation creep [23]
n_0	Stress exponent	1.0	3.5
A_0	Pre-exponential factor	1.0	9.0×10^{-20}
C_{OH}	OH concentration ($H/10^6$ Si)	1000	1000
r	C_{OH} exponent	1.0	1.2
E_0	Activation energy (kJ/mol)	335	480
V_0	Activation volume (m^3/mol)		
	Upper mantle	4.0×10^{-6}	11.0×10^{-6}
	Lower mantle	1.5×10^{-6}	-
d	Grain size (μm)		
	Upper mantle	10,000	-
	Lower mantle	40,000	-

Table S2. Parameters for all domains; related to STAR Methods.

Domains	Upper crust	Lower crust	Slab	Mantle	Accretionary prism
Density (kg/m^3)	2600	2900	3300	3300	2600
Initial viscosity (Pa·s)	1×10^{20}	1×10^{20}	1×10^{20}	1×10^{20}	1×10^{20}
Radioactive heat (W/m^3)	7.3×10^{-10}	1.4×10^{-10}	2.245×10^{-13}	2.245×10^{-13}	7.3×10^{-10}
for magnetic layers:					
oceanic	1.37×10^{-6}				
continental	2.0×10^{-6}				
Thermal conductivity (W/m·K)	2.5	2.5	2.5	2.5	1.4
for magnetic layers:					
oceanic	2.0				
continental	2.5				

References

1. Pollack, H.N., Hurter, S.J., and Johnson, J.R. (1993). Heat flow from the Earth's interior: analysis of the global data set. Reviews of Geophysics *31*, 267-280.
2. Ashi, J., Tokuyama, H., and Taira, A. (2002). Distribution of methane hydrate BSRs and its implication for the prism growth in the Nankai Trough. Marine Geology *187*, 177-191.
3. Ashi, J., Tokuyama, H., Ujiie, Y., and Taira, A. (1999). Heat flow estimation from gas hydrate BSRs in the Nankai Trough: Implications for thermal structures of the Shikoku Basin. Eos Trans. AGU *80*.
4. Tanaka, A. (2004). Geothermal gradient and heat flow data in and around Japan (II) Crustal thermal structure and its relationship to seismogenic layer. Earth, planets and space *56*, 1195-1199.
5. Tanaka, A., Yamano, M., Yano, Y., and Sasada, M. (2004). Geothermal gradient and heat flow data in and around Japan (I): Appraisal of heat flow from geothermal gradient data. Earth, planets and space *56*, 1191-1194.
6. Matsumoto, T. (2007). Terrestrial heat flow distribution in Japan area based on the temperature logging in the borehole of NIED Hi-net. pp. T23A-1217.
7. Qu, R., Ji, Y., and Zhu, W. (2021). Variations in Wedge Earthquake Distribution along the Strike Underlain by Thermally Controlled Hydrated Megathrusts. Applied Sciences *11*, 7268.
8. Müller, R.D., Sdrolias, M., Gaina, C., and Roest, W.R. (2008). Age, spreading rates, and spreading asymmetry of the world's ocean crust. Geochemistry, Geophysics, Geosystems *9*.
9. Siebert, L., Simkin, T., and Kimberly, P. (2010). Volcanoes of the World.
10. Omori, T., Watanabe, K., Umetsu, R., Kainuma, R., and Ishida, K. (2009). Martensitic transformation and magnetic field-induced strain in Fe–Mn–Ga shape memory alloy. Applied Physics Letters *95*, 082508.
11. Hacker, B.R., Peacock, S.M., Abers, G.A., and Holloway, S.D. (2003). Subduction factory 2. Are intermediate-depth earthquakes in subducting slabs linked to metamorphic dehydration reactions? Journal of Geophysical Research: Solid Earth *108*.
12. Grevemeyer, I., Diaz-Naveas, J.L., Ranero, C.R., Villinger, H.W., and Leg, O.D.P. (2003). Heat flow over the descending Nazca plate in central Chile, 32 S to 41 S: Observations from ODP Leg 202 and the occurrence of natural gas hydrates. Earth and Planetary Science Letters *213*, 285-298.
13. Li, C.-F., Lu, Y., and Wang, J. (2017). A global reference model of Curie-point depths based on EMAG2. Scientific reports *7*, 1-9.
14. van Keken, P.E., Wada, I., Abers, G.A., Hacker, B.R., and Wang, K. (2018). Mafic high-pressure rocks are preferentially exhumed from warm subduction settings. Geochemistry, Geophysics, Geosystems *19*, 2934-2961.

15. Wada, I., and Wang, K. (2009). Common depth of slab-mantle decoupling: Reconciling diversity and uniformity of subduction zones. Geochemistry, Geophysics, Geosystems *10*.
16. Brown, M., and Johnson, T. (2018). Secular change in metamorphism and the onset of global plate tectonics. American Mineralogist *103*, 181-196.
17. Penniston-Dorland, S.C., Kohn, M.J., and Manning, C.E. (2015). The global range of subduction zone thermal structures from exhumed blueschists and eclogites: Rocks are hotter than models. Earth and Planetary Science Letters *428*, 243-254.
18. Wang, K., Hyndman, R.D., and Yamano, M. (1995). Thermal regime of the Southwest Japan subduction zone: effects of age history of the subducting plate. Tectonophysics *248*, 53-69.
19. Iwamori, H. (1997). Heat sources and melting in subduction zones. Journal of Geophysical Research: Solid Earth *102*, 14803-14820.
20. Christensen, U.R. (1996). The influence of trench migration on slab penetration into the lower mantle. Earth and Planetary Science Letters *140*, 27-39.
21. Yoshioka, S., and Murakami, K. (2007). Temperature distribution of the upper surface of the subducted Philippine Sea Plate along the Nankai Trough, southwest Japan, from a three-dimensional subduction model: relation to large interplate and low-frequency earthquakes. Geophysical Journal International *171*, 302-315.
22. DeMets, C., Gordon, R.G., and Argus, D.F. (2010). Geologically current plate motions. Geophysical Journal International *181*, 1-80.
23. Hirth, G., and Kohlstedf, D. (2003). Rheology of the upper mantle and the mantle wedge: A view from the experimentalists. Geophysical monograph-american geophysical union *138*, 83-106.
